# Digenetic inheritance of *SLC12A3* and *CLCNKB* genes in a Chinese girl with Gitelman syndrome

**DOI:** 10.1186/s12887-019-1498-3

**Published:** 2019-04-18

**Authors:** Yuanmei Kong, Ke Xu, Ke Yuan, Jianfang Zhu, Weiyue Gu, Li Liang, Chunlin Wang

**Affiliations:** 1 The First Affiliated Hospital of Zhejiang University, Hangzhou, China; 2 Chigene Translational Medicine Research Center, Beijing, China

**Keywords:** Gitelman syndrome, Digenetic heritance, *SLC12A3*, *CLCNKB*

## Abstract

**Background:**

Gitelman syndrome (GS) is an autosomal recessive disorder and mild variant of classic Bartter syndrome. The latter is caused by defects in the genes *CLCNKB* and/or *CLCNKA* (chloride voltage-gated channel Ka and Kb). Patients with GS usually have loss-of-function mutations in *SLC12A3*. No patient has been reported with compound heterozygous mutations in these genes. We report a girl with GS with a paternally inherited heterozygous mutation in *SLC12A3*, and maternally inherited heterozygous variants in both *CLCNKB* and *CLCNKA*.

**Case presentation:**

In this report, we reported a female patient (8 y and 10 mo) who had growth retardation (111.8 cm, − 1.62 standard deviation height for age) and normal blood pressure, with persistent hypokalemia, hypomagnesemia, hypocalciuria, hypochloremic alkalosis, and elevated levels of plasma renin and aldosterone. Her younger brother, father, and paternal grandmother all had histories of mild low levels of plasma potassium (3.0–3.5 mmol/L), which were rectified by potassium-rich foods. The genomic DNA of the patient, younger brother, parents, and grandparents were screened for gene variations and pedigree analysis using trio whole exome sequencing (WES). The candidate variants were validated by Sanger sequencing. Protein-protein interaction analysis utilized the following databases: Biogrid, MINT, HPRD, STRING, IntAct, iRefIndex, and ppiTrim. The trio WES screening showed that the patient has paternally inherited *SLC12A3* p.N359K, and maternally inherited *CLCNKB* p.L94I. The paternal grandmother and younger brother are both carriers of *SLC12A3* p.N359K. According to the STRING database, SLC12A3 and CLCNKB proteins may interact or coexpress with proteins associated with GS.

**Conclusions:**

Based on clinical phenotypes, genetic evidence of the pedigree, and previous reported studies, this case of GS indicates a digenetic inheritance of *SLC12A3* and *CLCNKB* that resulted in renal tubular dysfunction perhaps, due to a genetic double-hit mechanism. The putative pathogenicity of the *CLCNKB* p.L94I variant requires confirmation.

## Background

Gitelman syndrome (GS, or familial hypokalemia-hypomagnesemia; Mendelian inheritance in man [MIM] #263800) is an autosomal recessive renal tubular salt-wasting disorder that is often characterized by potassium and magnesium depletion. Most patients with GS have loss-of-function mutations in *SLC12A3*, which encodes the thiazide-sensitive sodium-chloride cotransporter (NCC) [1, 2]. Some specific cases have putative mutations in chloride voltage-gated channel Kb (*CLCNKB*) [3–5] (ORPHA:358).

Clinical features of GS include transient periods of muscle weakness and tetany, abdominal pains, and chondrocalcinosis [1]. Asian patients with GS seem to have a greater tendency to hypokalemic paralysis than do patients of other ethnicities (approximately 6%) [6–8]. According to a study based on two unrelated Chinese families, female GS patients may have profound hypomagnesemia without neuromuscular symptoms [9].

GS is referred to as a mild variant of classic Bartter syndrome (or renal tubular normotensive hypokalemic alkalosis with hypercalciuria; MIM #607364), which is caused by defects in genes encoding chloride channel components, including *CLCNKB*, chloride voltage-gated channel Ka (*CLCNKA*), or both. Thus, previous studies focused on mutations in *SLC12A3*, *CLCNKB, CLCNKA,* and other genes associated with renal tubular functions.

Prior to the present, there has been no report of a case of GS associated with compound heterozygous mutations in the above genes. We report a girl aged 8 years and 10 months with GS, who has a paternally inherited heterozygous mutation in *SLC12A3*, and maternally inherited heterozygous variants in both *CLCNKB* and *CLCNKA*. This case may aid understanding of the phenotypic characteristics of the inactivated NCC and CLCNKA and CLCNKB in GS.

## Case presentation

The girl (aged 8 years and 10 months) was a full term baby delivered by planned natural birth. At age six years and seven months, she was found with growth retardation (111.8 cm, − 1.62 standard deviation [SD] height for age). She once presented at the local hospital with acute synovitis of the hip. Hypokalemia (2.93 mmol/L, reference: 3.5–5.3 mmol/L) for 2 months was incidentally discovered, but a further diagnosis was not made for the cause of hypokalemia. She had salt craving, thirst, polydipsia, and polyuria. She also complained of intermittent self-remitting tetany that occurred after prolonged activity and usually lasted for a few seconds. She denied other detrimental symptoms such as repeated episodes of fatigue, palsy, dizziness, hypotonia, palpitations, or blurred vision. After supplementation of oral potassium chloride 375 mg/day, her plasma potassium level reached 3.09 mmol/L. She still experienced recurring episodes of hand cramps, and for this reason was admitted to our hospital.

This girl was the first child of a non-consanguineous Chinese couple. She was not on any medications such as laxatives or diuretics, which could lead to a similar symptom. Her paternal grandmother had received a diagnosis of hypokalemia many years previously, at which time the plasma potassium level reached normal after a few days of spironolactone treatment. The girl’s father and younger brother also had been found with slight hypokalemia, and their plasma potassium levels reached normal after taking potassium-rich foods. Other pedigree members were all asymptomatic, and physical examinations were unremarkable.

On admission, the girl was alert, with no sign of anemia. Her vital signs were a temperature of 37.3 °C, pulse rate 92 beats/min, 20 breaths/min, normal blood pressure of 95/64 mmHg, and height 126 cm (− 1.39 SD for age). Her muscle tone and strength, and deep tendon reflexes were normal. The results of other physical examinations were negative.

Laboratory tests showed hypokalemia (3.33 mmol/L), hypomagnesemia (0.49 mmol/L, reference: 0.7–1.1 mmol/L), slight metabolic alkalosis (pH 7.43 and plasma bicarbonate 27.4 mmol/L, reference: 22–27 mmol/L), and hypocalciuria (0.01 mmol/L, reference: 0.48–5.87 mmol/L). The plasma aldosterone (216 pg/mL, reference: 59.5–173.9 pg/mL) and renin activity (13.43 ng/mL/h, reference: 0.15–2.33 ng/mL/h) were elevated on admission. Her thyroid function was normal and electrocardiogram revealed a normal sinus rhythm (95 beats/min) with normal QT interval. Abdominal ultrasound showed no renal stones or nephrocalcinosis.

The parents provided written informed consent prior to genetic testing. The genomic DNA of the patient proband, and her younger brother, parents, and grandparents were screened for gene variations and pedigree analysis using trio whole exome sequencing (WES). Briefly, the DNA was sheared and then hybridized with xGen Exome Research Panel v1.0 probe sequence capture array of IDT (Integrated Device Technology, USA) to enrich the exonic region. The exome libraries were first tested for enrichment by qPCR and for size distribution and concentration using an Agilent Bioanalyzer 2100. Exon-enriched DNA was sequenced on an Illumina HiSeq X Ten (Illumina, USA) platform in accordance with the manufacturer’s instructions. Raw image files were processed by BCL to FASTQ (Illumina) for base calling and generating the raw data. Low-quality variations of the quality score < 20 (Q20) were filtered out. The sequencing reads were aligned to the NCBI human reference genome (hg19) using Burrows-Wheeler Aligner (BWA) software. SAMtools and Pindel software were used to call single-nucleotide variants and indels of the reads. The minor allele frequency (MAF) was annotated using databases dbSNP, 1000 Genomes MAF (Chinese), ExAC, Genome Aggregation Database (gnomAD), and an in-house MAF database. Synonymous substitutions or single-nucleotide variants s with MAF higher than 5% were filtered out. Transcriptions and translations of nonsynonymous variants were predicted using SIFT (Sorting Intolerant from Tolerant; http://sift.jcvi.org), PROVEAN (Protein Variation Effect Analyzer; http://provean.jcvi.org), and PolyPhen-2 (Polymorphism Phenotyping v2; http://genetics.bwh.harvard.edu/pph2/).

The candidate variants were validated by Sanger sequencing and the pathogenicity of variants were annotated according to the American College of Medical Genetics and Genomics (ACMG) standards and guidelines. The UniProt database was searched for protein chain profiles of *SLC12A3* (ID P55017) and *CLCNKB* (ID P51801). Databases of known protein-protein interactions were used, including BioGRID, MINT, HPRD, STRING, and IntAct; iRefIndex data and its trimmed edition, ppiTrim, were both used to perform the interaction search.

**Fig. 1:**
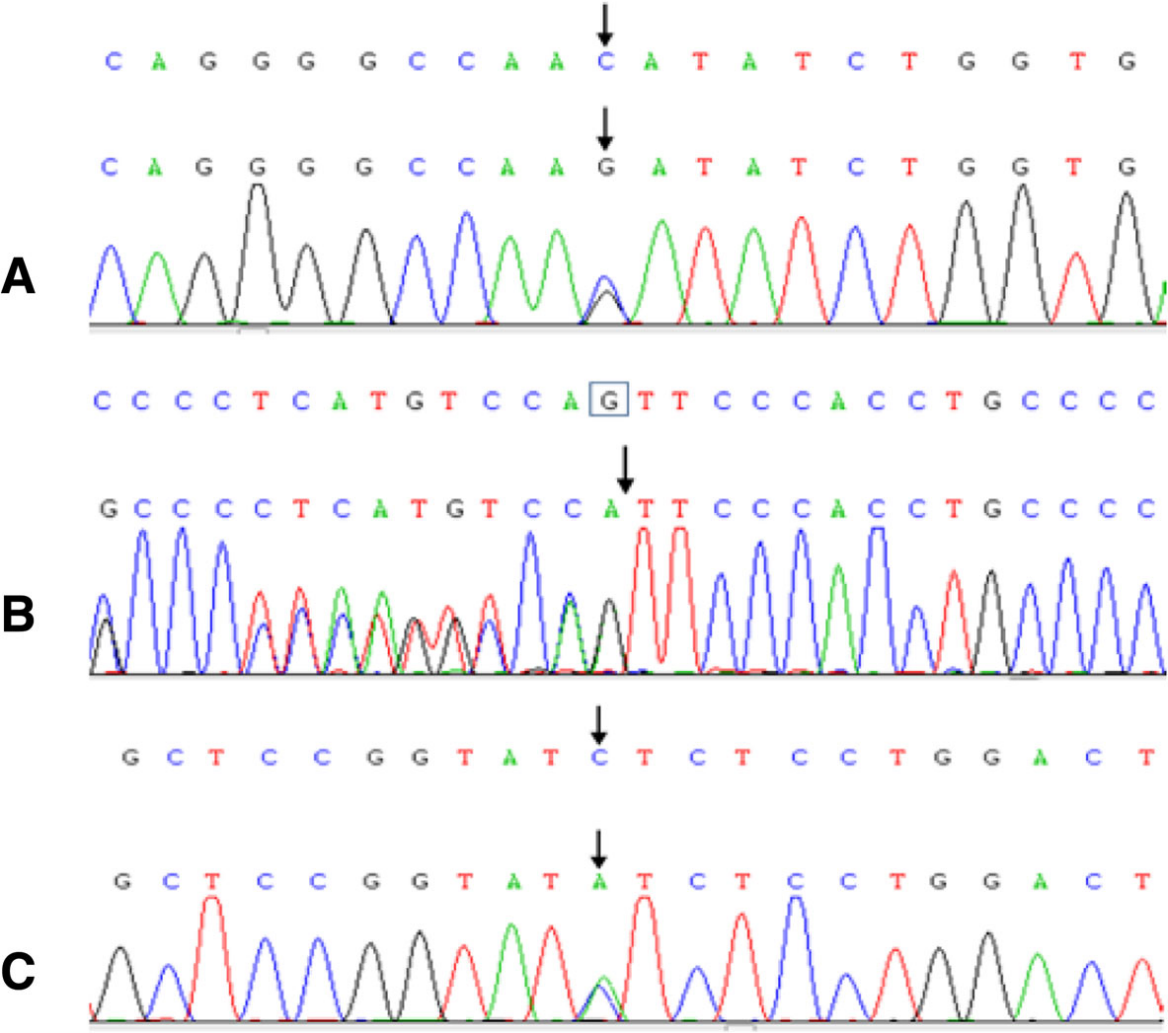
Sanger sequencing of candidate variants. (**a**) A paternally inherited heterozygous mutation was detected in *SLC12A3* p.N359K. In addition, maternally inherited heterozygous mutations were detected in (**b**) *CLCNKA* c.1054–22(IVS11)delG and (**c**) *CLCNKB* p.L94I

**Fig. 2:**
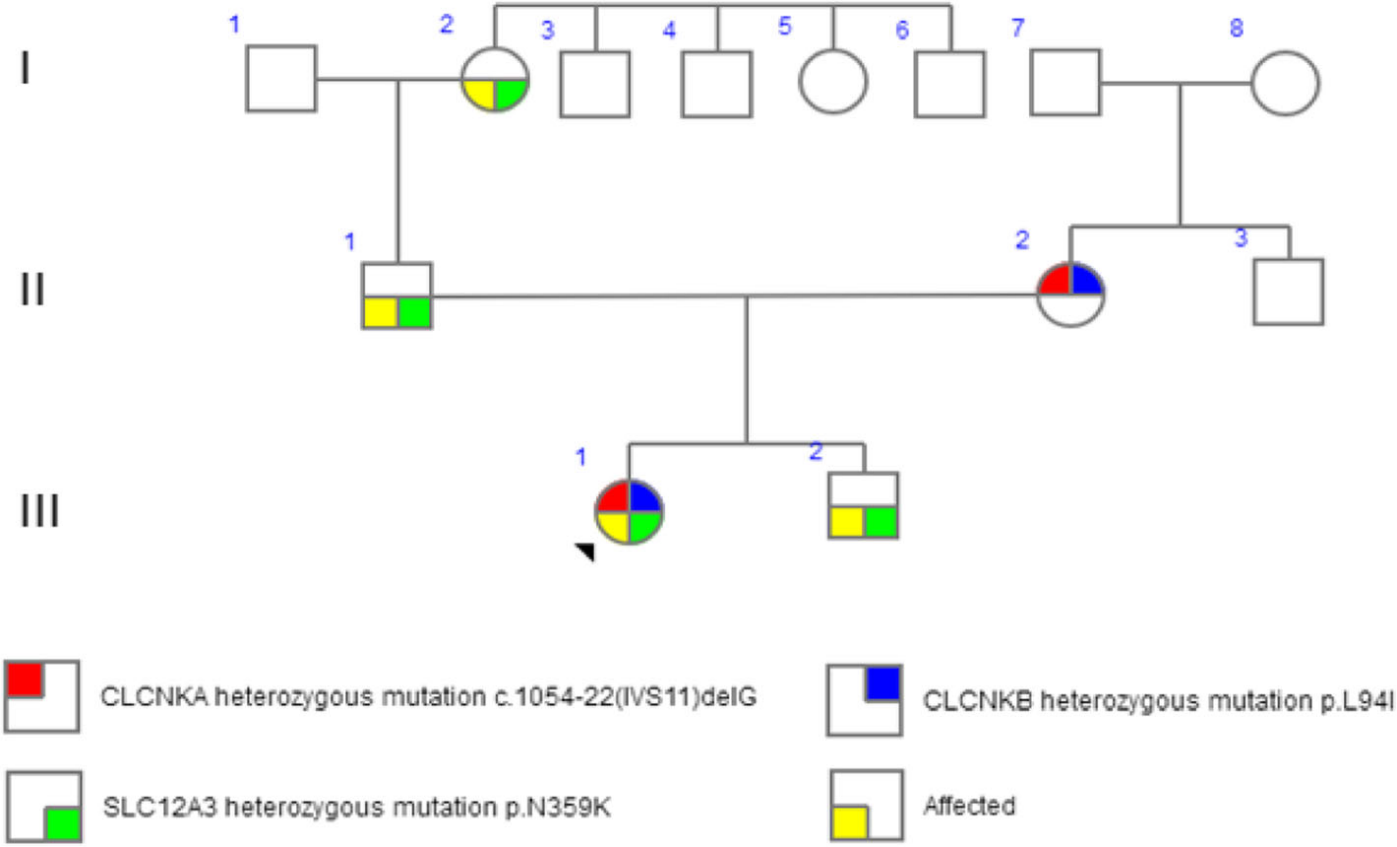
Pedigree chart of the proband’s family. The arrow indicates the proband. Men are indicated by squares, and women by circles. III1, the proband presents typical clinical manifestations and laboratory results of GS. I1, II1, and III2 present slight hypokalemia

Using trio WES, a paternally inherited *SLC12A3* p.N359K (NM_000339) and a maternally inherited *CLCNKB* p.L94I (NM_000085) were identified, and the variants were confirmed by Sanger sequencing (Fig. 1). The patient’s younger brother and grandmother were the carriers of *SLC12A3* p.N359K. No *SLC12A3* or *CLCNKB* variants were observed in other pedigree members (Fig. 2).

The *SLC12A3* p.N359K is a pathogenic variant according to the ACMG guidelines, with a low MAF < 0.005, and was previously documented pathogenicity [10]. According to the PM1 rating of the ACMG (i.e., located in a mutational hot spot and/or critical and well-establishedfunctional domain [active site of an enzyme] without benign variation), *CLCNKB* p.L94I is moderate evidence of pathogenicity. The functional predictions are conflicted, with M-CAP and mutation taster scores 0.1415 and 0.9956, respectively. This suggests that the variant is pathogenic. Yet, results of PROVEAN, SIFT, PolyPhen-2 HVAR, and PolyPhen-2 HDIV showed that the variant is tolerated. However, further research in the UniProt database revealed that *CLCNKB* (id P51801) p.94 is the first residue located at the second helix in the trans-membrane domain. All variants in *SLC12A3*, *CLCNKB*, *CLCNKA*, and the GS-related *HNF1B* genes were analyzed, and no other potential pathogenic variants were identified.

**Fig. 3:**
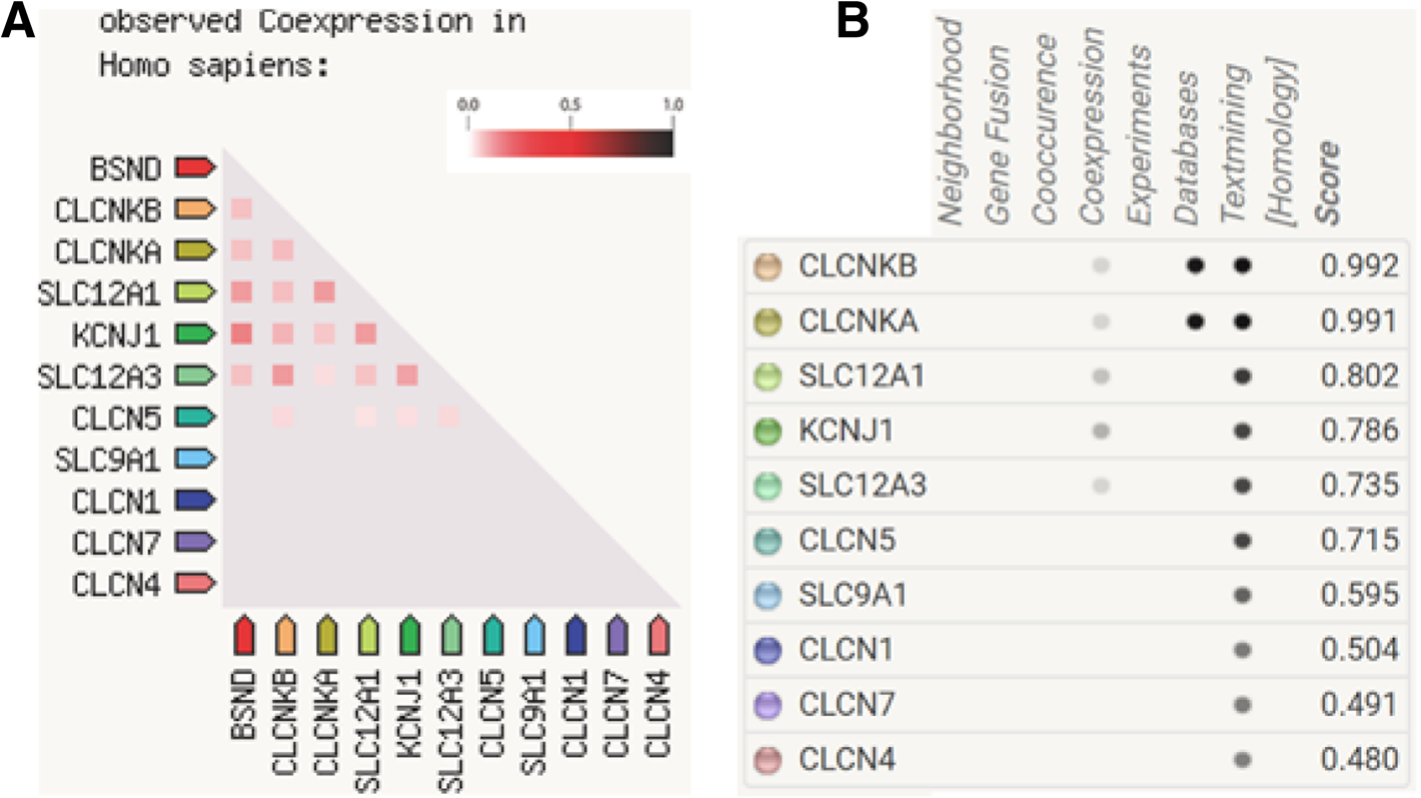
Protein-protein interactions in the Bartter syndrome network, according to STRING. (**a**) Protein-protein coexpressions. The gradient red bar showing the reliability rating. (**b**) Scores for evidence of the proteins included in the Bartter syndrome network

Using STRING tools, the protein-protein interaction analysis showed that *SLC12A3* and *CLCNKB* interact or coexpress in the human Bartter syndrome protein interaction network (Fig. 3).

## Discussion and conclusions

The diagnosis of GS was established on clinical rather than genetic grounds, potentially creating confusion with the related disorders classic Bartter syndrome and Bartter syndrome type III. Yet, phenotypic variability has also been documented in genetically confirmed GS patients, including those with identical *SLC12A3* mutations. A GS-like phenotype, with hypomagnesemia and hypocalciuria, has also been associated with pathogenic variants in the *CLCNKB* gene [8, 9]. The phenotype caused by *CLCNKB* mutations may vary, from the various types of Bartter syndrome to GS. Thus, a differential diagnosis is necessary.

As recommended by the latest KDIGO (Kidney Disease: Improving Global Outcomes) conference report, the next-generation sequencing panels for differential diagnosis of GS should include, at least, *SLC12A3*, *CLCNKB*, and *HNF1B* (hepatocyte nuclear factor-1-beta) [11, 12]. In the present case, a further genetic screening was conducted to exclude deletion or duplication in the *SLC12A3* allele (as previous reports have demonstrated [13]). Heterozygous variants in each of *SLC12A3*, *CLCNKA*, and *CLCNKB*, were confirmed.

According to previous studies, some using multiplex ligation-dependent probe amplification (MLPA) for screening, 15–20% of clinical GS patients have only one defect *SLC12A3* allele [11, 13]. For these findings, discovering pathogenic variants in other genes is recommended [11]. *SLC12A3* p.N359K was previously reported and pathogenicity was demonstrated by Qin et al [10]

The pathogenicity of *CLCNKB* p.L94I (rs201876924) has not been proved. The ACMG’s PM1 rating for *CLCNKB* p.L94I seems consistent with the function predictions. However, considering that CLCNKB is a membrane protein and the p.94 is at a trans-membrane domain, the deviation between the function prediction of the trans-membrane domain and other domains should be evaluated. Proving the pathogenicity of *CLCNKB* p.L94I needs further study.

The other suspect gene is *CLCNKA*. In the present case, a novel variant was found, a single base deletion that locates deep in intron 11 (c.1054–22 del G) of *CLCNKA*. Its pathogenicity was excluded using multiple prediction applications.

In the present case, there was no suspected variant of other GS-related or Bartter syndrome-related genes. In addition, no genotype-phenotype associations have been documented in patients with one heterozygous *CLCNKB* variant [14], which in our patient was inherited from the mother without such a condition. Thus, a digenetic inheritance (DI) may be involved in our case, since a defect in one allele of *SLC12A3* or *CLCNKB* would not cause the phenotypes.

In the present case, the clinical phenotypes of the paternal carriers indicated that heterozygous *SLC12A3* p.N359K may be responsible for transient and mild hypokalemia. However, this cannot explain the significant hypokalemia, hypomagnesemia, hypocalciuria, hypochloremic alkalosis, and elevated levels of plasma renin and aldosterone of the patient. On the other hand, compound heterozygous pathogenic variants in *CLCNKA* and *CLCNKB* can cause GS, which is considered a canonical pattern of DI. Interestingly, there was no sign of renal tubular dysfunction, and using bioinformatics, no potential pathogenic variant in *CLCNKA* was identified in the mother carrying the variants (Fig. 2). This helps to exclude the pathogenicity of *CLCNKA* c.1054–22 (IVS11) delG.

In summary, we identified a pathogenic variant in *SLC12A3*, a potential pathogenic variant *CLCNKB* p.L94I, and a likely benign variant c.1054–22 (IVS11) delG in *CLCNKA.* Since GS has been previously associated with several genes, we speculate that this case may represent a double hit pattern of DI.

Genetic linkage analysis is a reliable method of diagnosis, but has been relatively unsuccessful in identifying genes underlying human DI. In most reports of human DI, knowledge of candidate genes and protein-protein interactions have been used. Because finding ideal DI pedigrees is difficult, most studies base DI conclusions on a few patients without pedigree evidence. Schäffer [15] reviewed and evaluated three methods to identify DI genes, reporting that protein-protein interaction is important evidence. Protein-protein interaction analysis may be best for high-throughput data of variants and rare cases, even without a pedigree.

The coexpression and functional synergy of the proteins SLC12A3 and CLCNKB may be explained based on clinical phenotypes and physiological mechanisms. The results of the present case show that the coexpression of SLC12A3 and CLCNKB is stronger than the coexpression of SLC12A3 and CLCNKA (Fig. 3A), which agrees with the consensus [11]. The phenotype of patients with a CLCNKB defect usually overlaps with the GS phenotype, for example, hypokalemia and hypocalciuria [5, 16]. This may be explained by the localization of CLCNKB in the distal convoluted tubule. Interestingly, gender may influence the regulation of SLC12A3 function, based on a study from China [9] in which the hypokalemia of females with GS was less severe compared with the male family members. The authors speculated that this may be a positive effect of estrogen on *SLC12A3* or other modifying genes [9].

In this patient, GS was determined based on typical clinical data and the mutations *SLC12A3* p.N359K and *CLCNKB* p.L94I. We speculate that a digenetic mechanism led to the final phenotype. However, more evidence will be needed to confirm the pathogenicity of *CLCNKB* p.L94I, and further studies are particularly needed to explore the underlying molecular mechanisms.

## Abbreviations

ACMG: American college of medical genetics and genomics
DI: digenetic inheritance
GS: Gitelman syndrome
MAF: minor allele frequency
MIM: Mendelian inheritance in man
