# Correction to: Digenic inheritance of *SLC12A3* and *CLCNKB* genes in a Chinese girl with Gitelman syndrome

**DOI:** 10.1186/s12887-019-1705-2

**Published:** 2019-09-10

**Authors:** Yuanmei Kong, Ke Xu, Ke Yuan, Jianfang Zhu, Weiyue Gu, Li Liang, Chunlin Wang

**Affiliations:** 1 The First Affiliated Hospital of Zhejiang University, Hangzhou, China; 2 Chigene Translational Medicine Research Center, Beijing, China

**Correction to: BMC Pediatr**

**https://doi.org/10.1186/s12887-019-1498-3**

Following publication of the original article [1], the editor informed that error was found. The article describes “Digenetic inheritance” however, this needs to be corrected to “Digenic inheritance” as this is the correct term. The error is in the article title and several times mentioned in text (Abstract section under Conclusion section, page 4 and the abbreviation section).
